# Food Reward and Food Choice. An Inquiry Through The Liking and Wanting Model

**DOI:** 10.3390/nu12030639

**Published:** 2020-02-28

**Authors:** Almudena Recio-Román, Manuel Recio-Menéndez, María Victoria Román-González

**Affiliations:** 1 Tilburg University, Professor Cobbenhagenlaan 9 9.14, 5037 DA Tilburg, Brabant, The Netherlands; a.recioroman@tilburguniversity.edu; 2 Departament of Economy and Business, University of Almería, Carretera de Sacramento s.n., 04120 Almería, Spain; mvroman@ual.es

**Keywords:** consumer choice, self-control, taste, bundles, food reward, food choice, liking-wanting, obesity, variety-seeking, health

## Abstract

What if consumers are getting obese because eating less calories is more difficult for persons that have a higher pleasure and desire towards food (Ikeda et al., 2005) and food companies do not help given only a two extreme option choice to satisfy their needs (i.e., low calories vs. high calories or healthy vs. unhealthy)? Reward systems are being described with a new conceptual approach where liking—the pleasure derived from eating a given food—and wanting—motivational value, desire, or craving—can be seen as the significant forces guiding eating behavior. Our work shows that pleasure (liking), desire (wanting), and the interaction between them influence and are good predictors of food choice and food intake. Reward responses to food are closely linked to food choice, inducing to caloric overconsumption. Based on the responses given to a self-administered questionnaire measuring liking and wanting attitudes, we found three different segments named ‘Reward lovers,’ ‘Half epicurious,’ and ‘Non indulgents’. Their behavior when choosing food is quite different. Results show differential effects on caloric consumption depending on segments. The introduction of more food choices that try to balance their content is a win-win strategy for consumers, companies, and society.

## 1. Introduction

Obesity and overweight are critical global issues [1,2]. More than 2.1 billion people—nearly 30 percent of the global population—are overweight or obese. If the evolution of obesity continues on its current path, almost half of the world’s adult population will be overweight or obese by 2030 [3]. Even though almost two-thirds of the consumers are trying to lose weight [4], many of them fail in their goal [5,6]. Big food corporations’ marketing actions are one of the multiple causes of the problem [7,8,9,10]. The great variety, abundance, easy access, and high promotion of products that are perceived as more enjoyable, threaten caloric compensation plans followed by individuals [11,12,13]. This effect is even more significant for people that use food as a reward [14,15]. Our investigation shows how food pleasure—hedonic experience of eating [16]—and food desire—incentive motivation or disposition to eat [17]—, the two main components of food reward mechanisms, are positively related with the calories content in food choice and how a food bundle from two to five options mediates this relationship negatively. That is, when a consumer has a higher food pleasure or a higher food desire they are going to be more likely to choose the option higher in calories. This organic and intuitive relationship has been well studied [16,18,19,20,21,22,23,24]. However, to the best of our knowledge, an in-depth analysis on how the interaction between food pleasure and food desire works, and how this relationship influences food choice, has not been already done. It will also be seen that the more options are presented in the food bundle, the healthier choices consumers will make concerning the most caloric option. However, it will not work for all the consumer segments found. We shed light on when and why it happens. Furthermore, our study also shows that food desire is a stronger predictor of food consumption than food pleasure.

### 1.1. Problem Background

What if consumers are getting obese because eating fewer calories is more difficult for persons that have a higher pleasure and desire towards food [25] and food companies do not help given a two extreme option choice (i.e., low calories vs. high calories or healthy vs. unhealthy)? Consistent with this idea, we propose that any plan made by an individual to control the caloric intake will fail when the food reward system mechanisms are salient (i.e., food pleasure and food desire) and only the extreme choices of low caloric content and high caloric content are offered [1,26,27]. Therefore, instead of reducing the calories, the individual will be driven by the feeling of gratification—satisfaction that people derive from food consumption [28] leading to a surplus (eating over the caloric maintenance). It is necessary to align society and companies’ goals studying the different food-choice behaviors among consumers and offering tailor-made recommendations where the market approach is failing.

### 1.2. Relevance for Theory and Practice

#### 1.2.1. Relevance for Theory

Some studies have already discovered the relevance of food pleasure (liking) and food desire (wanting) when studying the different reward systems [18,19,29,30,31,32,33,34]. Furthermore, consistent with our previous idea, other studies have found a relationship between rewards systems with eating disorders such as binge eating [20,35], food addiction [1,36,37,38,39,40,41,42,43,44], obesity [14,26,45,46,47,48,49,50,51,52], and overeating [30,32,33,48,53]. Therefore, some studies suggest that reward systems contribute to eating behaviors, food choice, and food preference [1,19,54,55,56,57,58]. However, to the best of our knowledge, it is the first time that food choice is explained through the psychological traits of food pleasure (liking) and food desire (wanting) measured using existing self-administered general subscales—HTAS_pleasure for measuring food pleasure [59] and DBQ_emotional + DBQ_external to measure food desire [60]. Previously, these psychological constructs have been measured using a great variety of specific foods for knowing how food liking and food wanting have performed for each of them [61,62]. The subscales that we used implied an easier and more suitable instrument for managers and practitioners to measure and cope with the hedonic aspects of food choice. Hence, the construct operationalization adopted is different from the previous ones [61].

Additionally, our model proposes a vice-virtue bundle as a moderator. This idea has already been used in order to find a solution to help consumers manage choices between healthy and unhealthy food options [63]. Consistent with their concept, we will bring the novelty of seeing how two respondents with identical psychological traits concerning food pleasure and food desire, when either of those are high, will select a lower caloric content of food (healthier) when the vice-virtue bundle has five options than when they have two extreme options where the respondent will choose the most caloric one (unhealthier).

#### 1.2.2. Relevance for Practice

A discrepancy exists between the recommendations made by health organizations, companies, and the self-interest of consumers and actual food consumption. In order to better manage the consumers’ needs about food and its consequences at the individual, societal, and business level, it is essential to identify the factors that drive food choices [64,65].

For product marketing, it is crucial to segment consumers based on their attitudes to develop and produce food products that appeal to different groups with different attitudes and lifestyles [10,66]. From our study, it could be concluded that food pleasure and food desire are good predictors of caloric content related to food choice. Furthermore, this study will help to create more customized products according to the different psychological traits. In fact, the experiment of two versus five options has an extreme relevance because it will help to create plates that satisfy the customers’ choice without the need to increase calories. This experiment will help individuals to maintain their caloric intake (making them maintain their weight) or even in a deficit of calories (making them lose weight).

Successful food companies that carefully manage their customer base will obtain vital information to efficiently coordinate their long-run profit expectations and corporate social responsibility goals with complete customer satisfaction (McWilliams, Siegel, and Wright, 2006). Corporate social responsibility duties will be tackled thanks to the introduction of wider customized options. This will help to solve the problem of either food waste (because people do not eat everything that is on the plate due to caloric reasons) or obesity (because people eat everything that is on the plate surpassing their maintenance calories) [67]. This wider variety of choices will consist of giving the possibility to combine low caloric food (e.g., vegetables) with high caloric food (e.g., processed food) by dividing the plate into quarters.

Governments and Public Organizations will obtain valuable information to consider when regulating food marketing activities or launching social marketing campaigns over the diet-related health problems associated with the overconsumption of foods and beverages as, for example, the Harvard’s Healthy Eating Plate [68].

### 1.3. Theory, Hypotheses, and Conceptual Model

The individual understanding of what constitutes healthy eating has changed from having problems with gaining weight to face a daily battle in order to lose weight and try not to get it back [25]. Thanks to how food is marketed and labeled, our hedonic drives have been encouraged and exploited leading into a world where obesity and overweight are governing [64]. Thus, unraveling this hedonic drives may help us understand the factors that influence the excessive food intake associated with obesity [1,57,58].

Not only hedonic drives but also homeostatic ones guide food selection and intake. Both can be managed together to meet biological needs, i.e., eat something pleasant because you are hungry [29,64]. When this goal is reached, the hedonic drive can override homeostatic needs, leading to hedonic eating behaviors to satisfy psychological needs rather than physiological requirements, i.e., being full but end up in overeating because of pleasure [33,48,69]

Advances in neurobiology are helping to describe the structures that mediate the hedonic processes of consumption through the reward systems. Reward systems are being described with a new conceptual approach where liking—the pleasure derived from eating a given food—[18,19,33,34,70], and wanting—motivational value, desire, or craving—[16,33,34,57,71,72] can be seen as the major forces guiding human eating behavior [1].

Taking a more in-depth look into Consumer Behavior models, we could find Motivation, Opportunity, and Ability (MOA) [73] as a helpful tool for describing the different hedonic behaviors that people can carry through the learning process of information. Hedonic behavior is largely determined by stimulus incentive value, or its ability to function as a reward [74].

The creation and modification of this behavior are led by contingency learning (cognitive process). Contingency learning depends on motivational properties acquired by certain initially neutral stimuli during the contingency learning process [75].

This could be specifically explained by the Pavlovian system where the unconditioned stimuli (food attribute such as odor) create an incentive object (the food itself) that leads to a central motive state (eating the food). This positive appetitive unconditioned incentive stimulus becomes able to enhance the appetitive control motive state and promote effective approach responses (approach to eating) concerning the conditioned stimuli. This is relevant because the central motive state has neural processes that promote goal-directed actions in relation to particular classes of incentive stimuli such as food-seeking [75]. Furthermore, when the central route is used it is unlikely that the attitude will change and its more likely to predict future behavior [76].

When examining the role of food reward in eating behavior, one has to differentiate between food liking (pleasure) and food wanting (desire) [18,19,29,30,31,32,33,34,77]. By measuring these components separately, it is possible to learn under which circumstances they may differ by degree, helping to understand their role in food choice [19]. However, for achieving the full reward, the interaction between liking and wanting must be researched as well [1].

Taking a more in-depth look into how these variables may affect food choice, we can see how the three variables (liking, wanting, and the interaction) might have a positive effect on food choice (i.e., higher levels of liking, wanting, or both, leads to a higher calories content choice).

Higher levels of liking, wanting, and the interaction of liking and wanting, leading to a higher caloric consumption, could be explained through the reinforcement theory.

A reinforcer is a stimulus that increases the rate of a behavior that it follows. Strong reinforcers can motivate much behavior, whereas weaker reinforcers do not support very much behavior. The process of a behavior producing a positive consequence is also called reward learning [78]. When the reward is more effective, more reinforcement is needed to satiate that person (more high caloric food), and the more intense and persistent that person seeks reinforcement (compulsively seeking for caloric food) [79].

High caloric food can be seen as a strong reinforcer due to the substantial amount of behavior that people expend thinking about it and consuming it [79]. Food is a strong reinforcer, and, in some contexts, it may be a more powerful reinforcer than drugs [80].

Thus, more wanting and more liking of food will create a stronger food stimuli that at the same time will generate stronger responses for food consumption which will be reinforced by each time that the same behavior occurs (i.e., I eat a fast-food because I feel a high pleasure and the necessity to eat it; thus every time I can eat fast food against healthy food I will choose fast food, creating a habit).

Therefore, taking into account the theories mentioned above we hypothesized that, first, liking gives the motivational value of foods [56,81] and it is a determinant in range of foods eaten [82]. Therefore, increased food liking correlates with increased calorie consumption [27].

H1: Food pleasure (liking) increases caloric food choice.

Second, high food wanting is presumed to underline the excessive caloric intake [16,18,19,20,21,22,23,24] Liking and wanting seem to have different roles in overconsumption [62]. From the wanting perspective, there are some individuals that show an increased reactivity towards cues signaling the availability of food [83]. These susceptible individuals are characterized by higher levels of wanting which promote overconsumption in food rich environments [84]. Several studies [1,24,62,85,86] stated that repeated consumption of high palatable foods and drinks might develop neural sensitization that would come to experience exaggerated food wanting that is no longer in step with their food liking, promoting overconsumption and weight gain.

H2: Food desire (wanting) increases caloric food choice.

Third, even though ‘wanting’ is not ‘liking,’ both together are necessary for normal reward. ‘Wanting’ without ‘liking’ is merely a partial reward, without sensory pleasure in any sense. Several scholars have considered the independence of wanting and liking as a potential mechanism underlying a variety of human behaviors that negatively impact well-being such as overeating, pathological gambling, and the consumption of addictive substances. However, ‘wanting’ is still an essential component of normal reward, especially when combined with ‘liking’ tagging a specific behavior (i.e., consuming more calories) as the rewarded response [72].

H3: The interaction between food pleasure (liking) and food desire (wanting) increases caloric food choice.

Nevertheless, the incentive salience theory [87] established that wanting would be a stronger predictor of food consumption than liking. The incentive salience is the ability of the reward of the stimuli to trigger intense pursuit regardless of its hedonic quality temporarily. Therefore, we have to distinguish between incentive salience wanting (internal wanting) and ordinary wanting (external wanting) [61]. The latter is psychologically close related to explicit predictions of future value based on memories of previous pleasure on that outcome [64,88]. Incentive salience is not so rational [89]. Activation of incentive salience can cause people to intensely want things that they do not even expect to like very much [74]. Even it is possible to “want” what is not expected to be liked, nor remembered to be liked, as well as what is not actually liked when obtained [90]. Unexpected encounters with cues for a reward can activate motivation to pursue that reward as a goal. For instance, advertisements or the smell of a food near lunchtime make you quickly feel quite hungry. Indeed, several studies posit that “wanting,” more than “liking,” is an indicator of compulsive and problematic eating behavior. In other words, in the context of addiction, liking may stay relatively stable, or even decrease, as wanting increases [18,19,20,22,23,24].

H4: Food desire (wanting) will be more relevant in determining caloric food choice than food pleasure (liking).

Even though our research will follow the initial work of Berridge (1996), it needs to be pointed out that some researchers have refused this separation of concepts. On the one hand, some argue that liking and wanting cannot be separated (discerned). Claiming that all the previous works have been based mainly on animal studies, when looking into human rewards, wanting and liking are so intrinsically related that they cannot be considered as two distinct components having separate influences [17,91,92]. On the other hand, other researchers debate the independence between liking and wanting, declaring that liking influences wanting, and that wanting is lastly what influences food choice. The pleasure generated by food is probably determinant in defining what types of foods will become reinforcers [93].

Furthermore, wanting and liking can be increased due to cues triggering many choices context (Laibson, 2001; Lambert, Neal, Noyes, Parker, and Worrel, 1991; Stroebe et al., 2013) creating a problematic scenario for consumers where they struggle to not chose the unhealthy foods (high calories) (Shiv and Fedorikhin, 2002). Thus, given the opportunity to consume foods that are high or low in hedonic value, individuals will generally choose to consume foods that they find palatable consuming more calories as a result (Drewnowski and Hann, 1999).

Nevertheless, a solution for this issue was found by Liu, Haws, Lamberton, Campbell, and Fitzsimons (2015) called ‘Vice- virtue bundles.’ This solution was based on the idea that it is better to manipulate the quantity of unhealthy food (high in calories) by diminishing it rather than totally eliminating it [94,95,96]. It was due to the difficulty for the consumer to shift from pure vice—unhealthy and high in calories—to pure virtue—healthy and low in calories [97,98]. The quantities between pure virtue and pure vice can vary, such that a vice-virtue bundle might contain relatively more virtue (e.g., three tomatoes and one croquette), equal proportions of virtue and vice (e.g., two tomatoes and two croquettes), or relatively more vice (e.g., one tomato and three croquettes) against the extreme options of four tomatoes or four croquettes. Being the vice-virtue bundles the bridge between hedonic (wanting, liking or both) and health (moderate consumption of calories) goals, they allow the possibility of meeting both [63]. Accordingly, under the right circumstances where consumers are offered to reduce the quantity of pure vice, consumers may accept to increase the relative quantity of virtue instead (e.g., instead of eating four croquettes they chose to eat three croquettes and one tomato) [95].

This line of thought is based on the goal direct model. Taking into account the cognitive dissonance theory, which suggests that when people are presented with new information (a message) that conflicts with existing beliefs, ideas, or values, they will be motivated to eliminate the dissonance, in order to remain at peace with their thoughts [99]. This theory is further developed by the goal conflict model [100], where if a person has a diet goal and a hedonic goal, these two will conflict. Therefore, presenting more possibilities that primes the diet goal (healthier/virtue options) will lead the individual to select a more virtue option meeting the diet goal.

H5: The effect of food pleasure (liking) on caloric food choice is weaker when the vice-virtue bundle shows five options than when the vice virtue bundle shows two extreme options, leading to a negative relationship.

H6: The effect of food desire (wanting) on caloric food choice is weaker when the vice-virtue bundle shows five options than when the vice virtue bundle shows two extreme options, leading to a negative relationship.

H7: The effect of the interaction between food pleasure (liking) and food desire (wanting) on food choice is weaker when the vice-virtue bundle shows five options than when the vice virtue bundle shows two extreme options, leading to a negative relationship.

**Figure 1:**
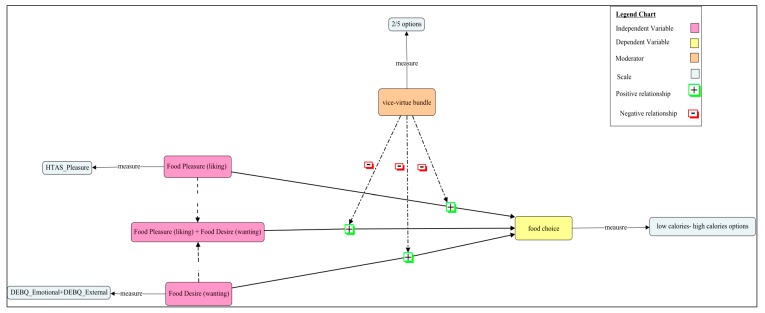
Food reward and food choice conceptual model.

Considering all the hypotheses, the conceptual model that we tested is depicted in Figure 1.

Nevertheless, it should also be taken into account that people have different set points [79]. Thus, some people will be more sensitive to food reward cues than others creating different level of preferences (i.e., some people will be hypersensitive to palatable food thus the diet goal will be eliminated selecting the pure vice option, some people will be more sensitive to diet cues selecting virtue options and some will not be sensible to food reward cues not presenting any goal conflict selecting the virtue options in every moment). This will probably generate different segments among the population, with the majority of them facing the dual conflict model.

## 2. Materials and Methods

### 2.1. Design

We use a between-subjects design to compare actual choice when vice-virtue bundles are included versus not included in a choice set. The primary purpose is to study whether the consumers’ choice among different vice-virtue bundles depends on food liking and food wanting attitudes.

### 2.2. Participants

Four hundred forty-eight students (61.9% females) at the University of Almería (Spain) participated in this study from mid to late November 2019. Forty-three of all participants were non-Spanish and non-eligible for our research. Of the four hundred and five remaining participants, one hundred and thirty-two respondents presented incomplete data. Hence, we obtained two hundred and seventy-three valid responses included in our analysis. While a convenience sample, it is representative of an important consuming segment (young people between eighteen and twenty-five years old).

### 2.3. Procedure

Data collection took place on mobile phones before the beginning of the classes, accessing trough an on-line Qualtrics questionnaire. Participants were informed that the survey they were going to answer was about how much food pleasure and food desire influence food choice. Next, they started to answer the questionnaire composed by the HTAS-pleasure subscale [59] (six items on a seven points Likert scale), the DEBQ-emotional, and external subscales [60] (twenty-three items on a five points Likert scale) and then, randomly, they were presented either a vice-virtue food bundle with two options, two plates of meal, or five options, five plates of meal. They had to choose the most preferred plate. It can be seen in Appendix A the different plate options that were presented. The two-option bundle consisted of asking consumers to choose between a low caloric option (a plate containing four slices of tomato) and a high caloric option (a plate containing four croquettes). The five-option choice set contained of all virtue (a plate containing of four slices of tomato), ¼ vice (a plate containing three slices of tomato and three croquettes), ½ vice (a plate containing two slices of tomato and two croquettes), ¾ vice (a plate containing one slice of tomato and three croquettes), and all vice (a plate containing four croquettes). The survey lasted around five minutes to be completed.

### 2.4. Measurements

The different variables (main and control) and the measurements of this survey research are described in Table 1.

### 2.5. Analysis Plan

First, we ran a descriptive univariate and bivariate analysis among the variables under study—associations between food liking, food wanting, two-option food set, five-option food set, BMI, and gender were tested using bivariate correlations.

Second, in order to test if the scales used for measuring food liking and food wanting were reliable enough, we computed the Cronbach’s Alpha and item to total correlations.

The next step consisted of knowing whether different segments existed among consumers attending to their food liking and food wanting attitudes. To test it, we ran a hierarchical cluster analysis, standardizing the data previously because they were measured on different range scales. We used the Euclidean distance—most commonly used measure of the similarity between two objects—and the Ward’s method—this method tends to result in clusters of approximately equal size due to its minimization of within-group variation [102].

Once we found the segments, we checked if their food choice behavior differed, analyzing the mean differences through an ANOVA analysis. Once converted the food options to calories, we checked whether when more options were available (5 options vs. 2 options), people that belonged to the found segments reduced their caloric intake.

Finally, in order to take a more in-depth insight into how much food liking, food wanting, and the interaction between them influenced the food choice option made by the respondents, we used a logit (two-option food choice set) and a multinomial regression (five-option food choice set). We analyzed the interaction term calculating the marginal effects to see how wider food choice bundles affected the results. Finally, for corroborating the obtained results we used a non-linear technique, Classification and Regression Trees (CART). Furthermore, we obtained practical results that would allow, using the food liking and food scores, to predict the food choice that people will do. For generating robust results, we trained the algorithm using a repeated 10-fold cross-validation.

## 3. Results

### 3.1. Descriptives

Descriptive statistics and correlations among all variables are shown in Table 2. Food liking and food wanting are significantly correlated (r(271) = 0.56, *p* < 0.01). Both of them have significant correlations with the food choice scales used, being lower in the case of food liking (r(136) = 0.22, *p* < 0.05 and r(133) = 0.51, *p* < 0.01)) in comparison with food wanting (r(136) = 0.46, *p* < 0.01 and r(133) = 0.77, *p* < 0.01). BMI has no significant correlation with the other variables under study. Gender has low and significant correlations with food liking (r(271) = 0.12, *p* < 0.05) and food wanting (r(271) = 0.16, *p* < 0.01).

In Table 3 we depict the percentage of participants that selected each of the options presented across study conditions. In the pure virtue-pure vice choice set, the choice shares were not significantly different (observed proportion = 0.58, z = 1.90, *p* = 0.07), selecting 58% the pure virtue option and 42% the pure vice one. In the vice-virtue bundle with five options, 22% of respondents chose pure virtue, 32% chose the ¼ vice option, 29% chose the ½ vice option, 14% chose the ¾ vice option and 2% chose pure vice. Pure vice option was significantly less chosen that the other options (observed proportion = 0.04, z = −9.49, *p* < 0.01). It is also remarkable that the ¼ vice was the most chosen alternative, being significantly different from the pure virtue option (observed proportion = 0.65, z = 3.65, *p* < 0.05), and the ¾ vice option (observed proportion = 0.66, z = 3.92, *p <* 0.03).

Results indicate that people elect central vice-virtue bundles when making a food choice and not only the extreme options. In Table 3, we notice that in the aggregate, the central choice options (¼ vice, ½ vice, and ¾ vice) are preferred to the extreme options (pure virtue and pure vice). We need to link these results with the different consumer segment profiles formed attending to food liking and food wanting criteria.

### 3.2. Scales Reliability Analysis

In order to measure food liking, we used the HTAS-pleasure subscale composed of six items. For measuring food wanting, we used the DEBQ-emotional and external subscales. For all of them, we ran a reliability analysis. Table 4 shows that the scales used are reliable enough to work with them (α_HTAS_pleasure_ = 0.74, α_DEBQ_emotional_ = 0.93 and α_DEBQ_external_ = 0.74). In addition, the main descriptives are available in Table A1 (Appendix B).

### 3.3. Segmentation

When selecting a bundle option, consumers face a trade-off between health and taste [63]. This reasoning could also be translated to the food liking and food wanting paradigm. The hedonic motivation felt by a consumer towards the food (liking) and the experienced sensation of a lack of something desirable or necessary provoked by an incentive salience attribution (wanting) could be moderated by the individual susceptibility to overeat [33]. In order to evaluate how processes of ‘liking’ and ‘wanting’ influence on food-choice behavior, it is relevant to study the heterogeneity that exists across consumers.

Cluster analysis is the name of a group of multivariate techniques whose primary purpose is to classify a sample of objects (individuals in our case) into a small number of mutually exclusive groups based on the similarities among the individuals. These groups are usually called clusters. Each different group formed contains persons that are more like one another than they are like persons in a different group [102]. When the groups are not predefined is one of the best techniques to use [103]. In order to analyze the heterogeneity that exists across consumers, we perform a cluster analysis using the sum score scale of HTAS_pleasure (liking) and the sum score scale of DEBQ_emotional + DEBQ_external (wanting). We ran a hierarchical cluster in IBM-SPSS Statistics version 25, standardizing the scales previously, applying the Euclidean distance and the Ward method.

**Figure 2:**
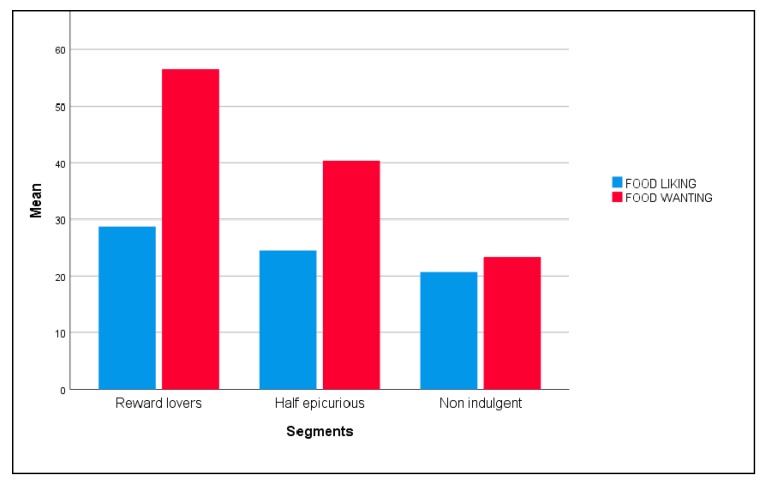
Segments profiles based on food liking and food wanting (means).

**Figure 3:**
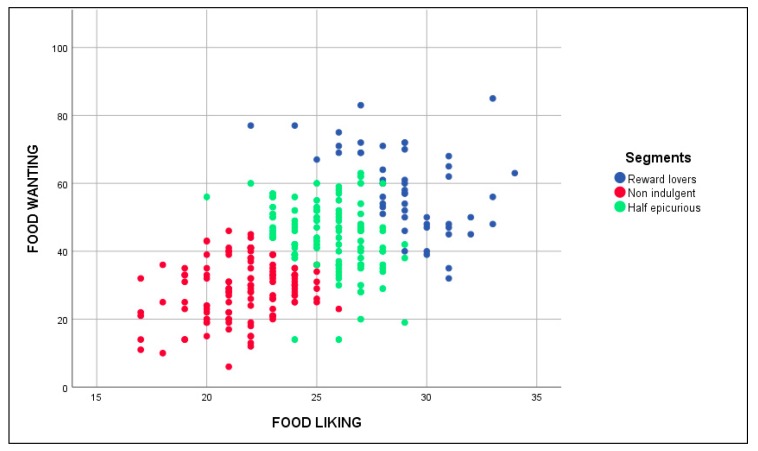
Segments positioning based on food liking and food wanting total scores.

Looking at the resulting dendrogram (see Appendix C), we concluded that three different segments existed. Figure 2 shows their profiles using the food liking a food wanting average scores and Figure 3 paints the positioning of the segments, and each of the respondents, according to their food liking and food wanting total scores. We label the first segment as ‘Reward lovers’ (*n* = 63, 23.1% of the total sample). They have the highest scores on the liking and wanting scales. We expect that their profiles are going to be closely connected to those that Liu et al. (2015) called ‘Vice lovers.’ Following the ‘unhealthy = tasty’ intuition [104] they are expected to perceive vices to be tastier than virtues, choosing pure vice over pure virtue in the absence of vice-virtue bundles, suggesting that ‘they may place a higher importance on addressing a taste goal than a health goal when both cannot be addressed simultaneously’ [63]. Subsequently, we predict that when they face the bundle with five options, they select the alternatives with higher vice content (½ vice, ¾ vice and full vice). In these alternatives, the presence of some virtue in the plate allows them to calm their guilty conscience and reduce the goal conflict, causing the options with more virtue content (full virtue and ¼ vice) not so much elected.

We named the second segment as ‘Half epicurious’ (*n* = 142, 52.0% of the total sample). They are situated halfway between the two extreme segments in food liking and food wanting terms. For that reason, we expect a balanced result when they have to choose between the two extreme food options and sparing their selection towards the central possibilities (¼ vice, ½ vice, and ¾ vice) in the five options bundle. We presume that their profile will also match with the one explained by Liu et al. (2015) in the case of the ‘Virtue acceptors’ segment. For the people that belong to this segment, they place greater importance on addressing a health goal than a taste goal. Therefore, it is likely that they would prefer a vice-virtue bundle with a lower vice proportion in comparison with the ‘Reward lovers’ segment.

We called the third segment ‘Non indulgent’ (*n* = 68, 24.9% of the total sample). We suppose that they match with the profile named ‘Virtue lovers’ by Liu et al. (2015). Hence, they prefer the result of more virtuous options because, through them, both taste and healthy options can be achieved. When the five plate options bundle is presented to them, we expect that one of the main reasons to introduce more vice in their election would be variety seeking [105,106,107]. Therefore, they will select the options with more virtue (full virtue, ¼ vice, and ½ vice). The options with more vice content will be much less elected simply because the goal conflict arises.

**Figure 4:**
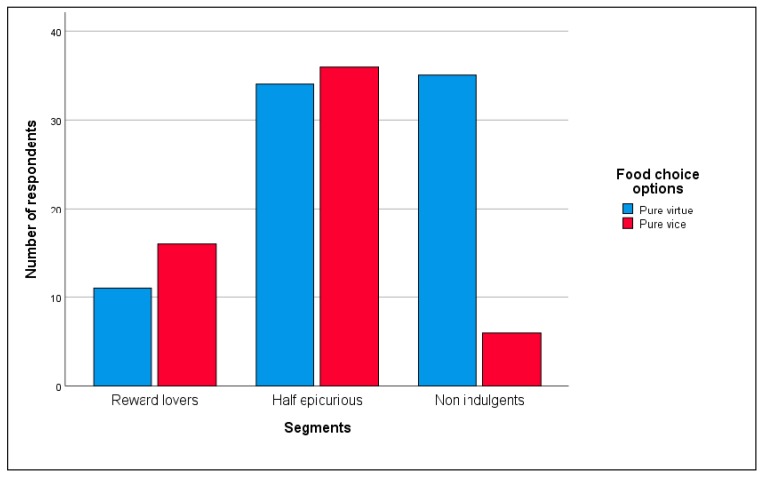
Segment’s food choice. Pure virtue-pure vice set (number of respondents).

**Figure 5:**
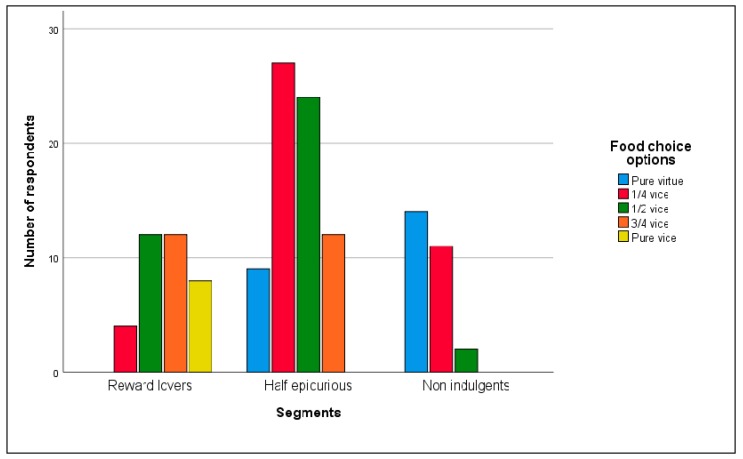
Segments food choice. Five options bundle (number of respondents).

Figure 4 and Figure 5 (Table A2 and Table A3 in Appendix D) illustrate the alternatives chosen by each segment.

From Figure 4 (Table A2 in Appendix D), we see that 60% of ‘Reward lovers’ preferred the pure vice option but not at significant levels (observed proportion = 0.6, z = 1.06, *p* = 0.44). Additionally, 85.4% of the ‘Non indulgents’ chose the pure virtue option (observed proportion = 0.85, z = 6.28 *p* < 0.01) and 48.6% of the ‘Half epicureans’ selected the pure vice option but not at significant levels (observed proportion = 0.49, z = −0.16, *p* = 0.9). Hence, the previously expected segments’ food choice behavior profile hold for ‘Non indulgents’ and ‘Half epicureans’ but not for ‘Reward lovers’ at significant levels.

From Figure 5 (Table A3 in Appendix D), we observe that for ‘Reward lovers’ the most preferred options are those with higher vice components and none of the respondents selected the pure virtue option. These entire three alternatives (½ vice, ¾ vice and pure vice) taken together are significantly different from the two less vice content ones (observed proportion = 0.89, z = 7.84, *p* < 0.01). We find the opposite situation for ‘Non indulgents.’ None of them picked the pure vice option or the ¾ vice option. They only favored food choice alternatives with less vice content (pure virtue, ¼ vice, and ½ vice). For ‘Half epicurious’ the most chosen options were the central ones (¼ vice, ½ vice, and ¾ vice) being the ¼ vice option significantly preferred to the ¾ vice option (observed proportion = 0.69, z = 3.34, *p* = 0.02). Consequently, predicted behaviors for each of the segments mostly holds.

In Table 4 and Table 5, we see the food choices made by respondents transformed into calories. These calories have been calculated through examining the label of the croquettes package (175 kcal per croquette) and multiplying them depending on the number of croquettes in the plate, plus summing the calories of the tomatoes in the same way (taking into account that the calories of an average Kumato tomato of 150 g are 31 kcal). The introduction of vice-virtue bundles slightly reduces the aggregate caloric consumption (−3.6%). Results show differential effects on caloric consumption depending on segments. ‘Non indulgents’ increase a little bit the calories elected (+2%), ‘Half epicurious’ reduce the caloric selection made (−9.3%), and ‘Reward lovers’ increase the calories chosen (+12.6%).

**Figure 6:**
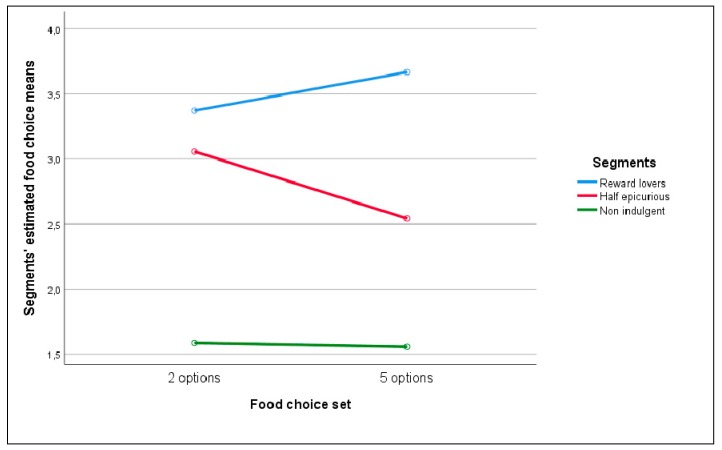
Mean food choice selection as a function of segment and food-choice set.

In order to analyze the mean food choice difference among segments a 2 (food choice: 2 options vs. 5 options) x 3 (segments: ‘Reward lovers’ vs. ‘Non indulgents’ vs. ‘Half epicureans’) ANOVA analysis was run. As is clear from Figure 6 and Table A4 (Appendix E), there is only a significant main effect of segments on food choice F(2267) = 29.48, *p* < 0.01), indicating that the vice- virtue composition of the selection made by the respondents was significantly higher for ‘Reward lovers’ (M = 3.52, SD = 0.86) and ‘Half epicurious’ (M = 2.80, SD = 0.12) than for ‘Non indulgents’ (M = 1.57, SD = 0.18). No other effects were significant (There was not a significant interaction between segments and food choice options (F(2,267) = 1.83, *p* = 0.162)).

From Figure 6, we also observe that the unexpected change that we already saw when analyzing the caloric choices made by segments in Table 4 and Table 5 was clearly represented. When introducing more options in the food bundle, we supposed that, for all segments, the vice content of the choice made would be reduced. However, it was not true neither for ‘Rewards lovers’—they increased the calories consumed—nor for ‘Non indulgents’—they practically maintained the calories consumed. At this point, we needed to check the robustness of this counterintuitive result coming back to our food liking-food wanting variables in order to see how they behaved when respondents were choosing the different food alternatives available. Segments were built considering the similarities and differences in food liking and food wanting expressed by respondents when answering the self-administered subscales. Nevertheless, the interaction effects were not taken into account. These interactions are meaningful because several studies stated that a clear distinction exists between how pleasant or painful a stimulus event will be (liking) and the interaction between the stimulus event and the current concerns of the individual perceiving it that determine incentive salience or wanting [108,109,110].

### 3.4. Food choice Explained through Food Reward (Food Liking and Food Wanting)

In favor of knowing how much the choice between different food options varies depending on food liking, food wanting, and the interaction between food liking and food wanting, we performed a logit analysis (food choice set with 2 options) and a multinomial analysis (food choice set with 5 options). From the results in Table 6, we conclude that for the two-options set, only food wanting and the interaction between food liking and food wanting significantly influence food choices made by interviewees. In the two choices set, each point of increment in food wanting rise a 37% the probability of selecting the pure vice vs. pure virtue option. In the five choices set, food liking, food wanting, and the interaction between them are significant (exception made for food wanting in the pure vice option). Hence, for every one-unit increase in food wanting increases a 42% the probability of choosing ¼ vice vs. pure virtue, a 54% the chance of choosing ½ vice vs. pure virtue, a 67% the likelihood of choosing ¾ vice vs. food virtue, and a 97% the odds of selecting pure vice vs. pure virtue. In the case of food liking, for every one unit increase rises a 41% the probability of choosing ¼ vice vs. pure virtue, a 55% the chance of choosing ½ vice vs. pure virtue, a 53% the likelihood of choosing ¾ vice vs. food virtue, and a 29% the odds of selecting pure vice vs. pure virtue. Interaction effects are significant for all the options presented. Interpretation of interaction effects in logit and multinomial regressions are easier to understand when using marginal effects. For interpretation purposes, marginal effects are popular because they make available the amount of change in the dependent variable (Y) that will be given by a 1-unit change in an independent variable (X), while all other variables in the model remain constant [111]. Hence, they are easier and more intuitive to interpret than in odds or log-odds terms. There are three different marginal effects approaches: marginal effects at the means (MEMs), average marginal effects (AMEs), and marginal effects at representative values (MERs). Following Bartus’ (2005) [112] recommendations, we used MERs because it allows choosing a range of values for one or more independent variables, focusing on how marginal effects behave in that range (in our analysis it was studied how interaction effect affected food choice at each level of food liking).

**Figure 7:**
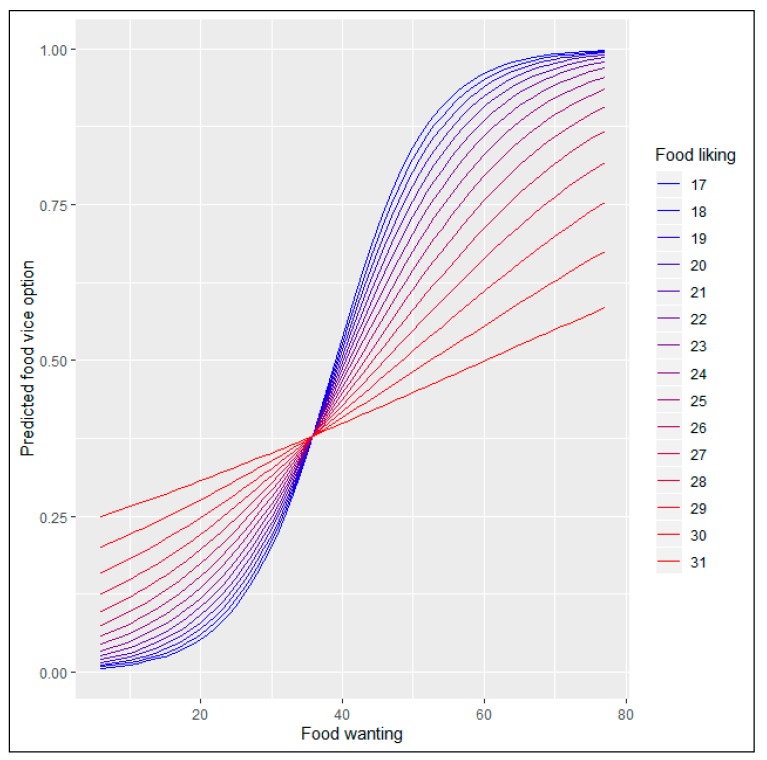
Marginal effects of the interaction term (Food liking*Food wanting) in the two options set’s logit regression.

**Figure 8:**
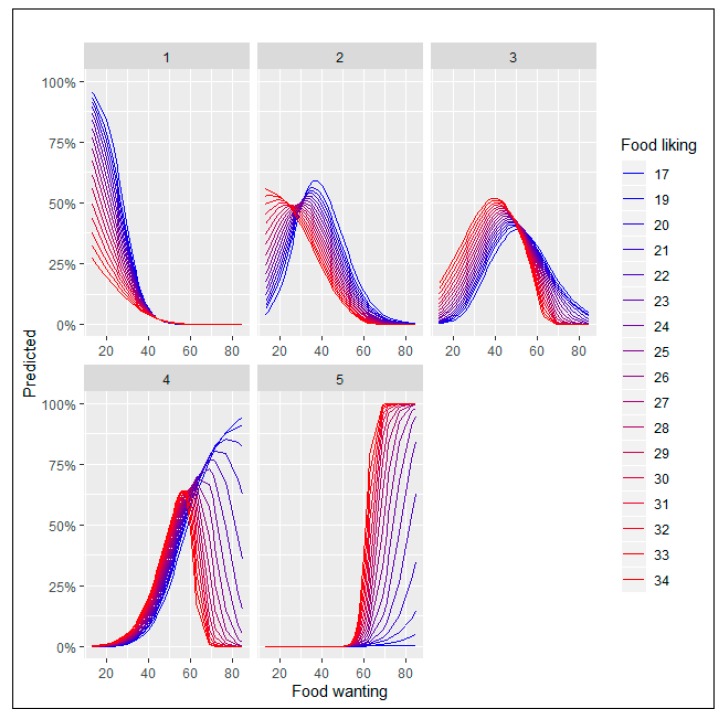
Marginal effects of the interaction term (Food liking*Food wanting) in the five options set’s multinomial regression.

Figure 7 and Figure 8 depict the results for the marginal effects of the interaction terms found in the logit and multinomial regressions.

We see in Figure 7 that the interaction effect between food liking and food wanting on choosing the pure vice option, when only the two extremes alternatives are present, depends on the levels of both of them. We also observe that the shape of the relationship changes from a rectilinear form, when the food liking is high, to an S-shaped curve when the food liking is getting lower. The evolution from one relationship shape to the other perfectly follows the escalation of the food liking levels. We define incentive sensitivity as the change produced in the probability of choosing one food-choice option for every unit change in the food wanting scale. Using this concept, we observe that lower liking levels (the ones with S-shaped curves) have, when food wanting is low, a lower incentive sensitivity than the higher liking levels (the ones with a rectilinear or quasi-rectilinear shape). When food wanting scores 20, the marginal incentive sensitivity raises sharply for the S-shaped curves maintaining a high slope until food wanting points 50. Graphically we see that the different slopes of the marginal effects functions reach a common point when food wanting equals 36. From levels of food wanting greater than 36, we observe that the probability of choosing the pure vice option is higher for people whose food liking is lower. As we explained before, it is due to the higher incentive sensitivity that they comparatively have.

Figure 8 depicts the marginal effects of the interaction between food liking and food wanting when the consumer faces the five option food choice set.

Plot 1 shows that people with lower levels of food liking are more likely to elect the pure virtue option for each level of food wanting. Furthermore, for scores of food wanting greater than 40 points, the probability of choosing this alternative is almost zero.

Plot 2, ¼ vice option, displays a turning point (maximum) when food wanting is 28 points and above. In all the cases, higher levels of food liking need less food reward to obtain the same probability of selecting this option. It means that when the expected pleasantness (liking) for a consumer is higher, the strength of the incentive salience for rewarding (wanting) is lower in order to have the same probability of choosing the ¼ option. In simple terms, when liking is higher, the incentive needed to choose this option is lower. Graphically it is easy to observe when crossing a horizontal line at any level of the predicted probability: when lines are more in red (higher liking), the projected points on the *X*-axis (wanting) will have a lower value. It is also interesting to point out that the maximum probability of selecting this option is for people that have the lowest reported food liking (17 points) when food wanting reaches 40 points. It seems logical that people with lower levels of food liking will more likely elect this option with low vice content. Furthermore, we also notice that when food liking is in the surroundings of 30 points, lines representing each liking level invert their relative position with respect to the other levels. From this point to the left, lines that represent higher levels of food liking are always over the lower levels and vice versa from this point to the right of the plot. It is also remarkable that the different shapes of the lines depend on the food liking level: higher levels present an inverted S-shaped curve and gradually become the form of a bell-shaped curve as the food liking levels decrease. In behavioral terms, it is plausible that people with higher levels of food liking show an always-decreasing probability to choose an option with a low vice content. In other words, the more the food liking the less probability to select the low vice content alternative (inverted S-shaped curve); as food liking level decreases, the incentive to select this option has to increase until it reaches a maximum. Passing this turning point (maximum probability to pick this option), every increase to incentive this option will reduce the probability of being chosen. In practical terms, it also has a consequence when managing this food choice option (¼ vice): for each level of liking, there is an optimal point of wanting to reach the maximum probability to be selected. For people with higher levels of food liking the inverted S-shaped curve also means that the more spontaneous the response (less incentives or cues to promote it) the higher probability to prefer the option with only ¼ vice content. It also occurs in plot 1 (pure virtue option), where we can observe that the highest probabilities to choose this option occurs when food wanting is at their minimums. In the end, it would mean that for people with higher food liking profile (‘reward lovers’ segment in our case), when they have more options to pick from (five options), the more virtue content alternatives would be less selected than when they have fewer alternatives (two options).

Plot 3, ½ vice option, exhibits a bell-shaped relationship with a turning point (maximum) when food wanting scale is 50 points. In all the cases, higher levels of food liking need less food reward to obtain the same probability of selecting this option. Again, the reason why it happens is the same as we already explained in Plot 2: as liking is higher, the incentive needed to pick this option is lower. In this case, all are bell-shaped curves. The change of the shape for the people with higher levels of liking could be explained through the greater vice-content of this alternative. This option is more attractive for them, and the probability of choosing it always needs less incentive (wanting). Maximum probabilities to be selected the half-vice option are reached for the higher-liking people in the surroundings of 35-37 points of the food wanting scale.

Plot 4, ¾ vice option, pictures a turning point (maximum) when food wanting scores 58 points. Below that level, the ascendant part of a bell-shaped curve, the persons with greater levels of food liking have a higher probability of picking this option. Above that level, people with greater food liking punctuations follow the descending part of the bell-shaped curve, but persons with the lower food liking scores increase the probabilities of choosing this option when food wanting raises (the lowest the food liking level the highest the probability of choosing this option). As we already explained for the two-options food choice set, a different incentive sensitivity seems to be present.

Plot 5, pure vice option, shows the inverse situation to the one pictured in the pure virtue plot. Until the food wanting score reaches the 50 points, the probability of choosing this option is almost zero. Above this level, people with a greater food liking experience a sharp rise in the likelihood to choose this alternative with each increasing unit of food reward. For those with the lower levels of food wanting the probability to select this alternative is very low.

In summary, we found different incentive sensitivity when analyzing the interaction effect of food liking and food wanting on the likelihood to choose the pure vice option in the food choice set with two options. Lower food liking persons have a higher incentive sensitivity for most of the combinations. It means that all the marginal effects lines that represent the marginal effects for each level of food liking converge to the same probability to choose the pure vice option when food wanting reaches 35 points. From this point onwards, people with lower scores on food liking are more likely to select the food vice option at each level of food wanting. When the vice-virtue bundle with five options is presented, the relationship changes. The probability of selecting the pure vice option is almost zero until the food wanting score reaches the 50 points. From this point, the higher the food liking and the food wanting scores the higher the likelihood to choose the pure vice option. Nevertheless, the ¼ vice, ½ vice and ¾ vice options present different characteristics. In the two first cases, the shape of the relationship is different from the one found in the two options food set for the pure vice alternative. We found a bell-shaped curve for most of the cases and not an S-shaped curve. It means that for the ¼ vice and ½ vice options, the probability to be chosen decreases when the turning point (maximum) is reached. For the ¾ vice option, a mixed situation was found: people with greater food liking levels follow a bell-shaped curve and people with lower food liking levels portrait an S-shaped curve. Hence, we found some similarities for the people with lower food liking scores in the pure vice option for the two options food choice set and the ¾ vice alternative for the five options vice-virtue bundle.

**Figure 9:**
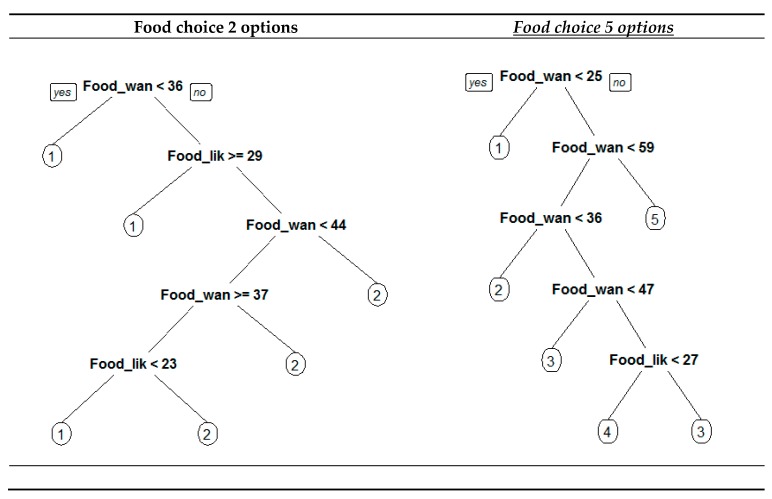
Classification tree food choice—2 and 5 options.

Finally, we would like to check how food liking and food wanting predict choices when nonlinear techniques are applied. We used Classification and Regression Techniques (CART) to accomplish this goal. For generating robust results, we trained the algorithm using a repeated 10-fold cross-validation in order to optimize the model complexity parameter (RMSE was used to select the optimal model using the smallest value (2 options-> cp = 0.15, 5 options-> cp = 0.02). Figure 9 depicts the results obtained. In both trees, we see that food wanting is the main criteria used to split the respondents into the different options available. The classification trees obtained, reached an accuracy of 63.3% in the 2 options food choice set and 71.06% of the 5 options food choice set. In other words, using the models shown in Figure 9, we correctly predict the election made by the consumers in the percentages mentioned before. From the practical perspective, it gives a useful tool for predicting consumer behavior in relation to food choice: knowing the scores given by the respondents to the subscales used and the heuristics rules derived from the CART results, we can predict which food choice they will select.

**Figure 10:**
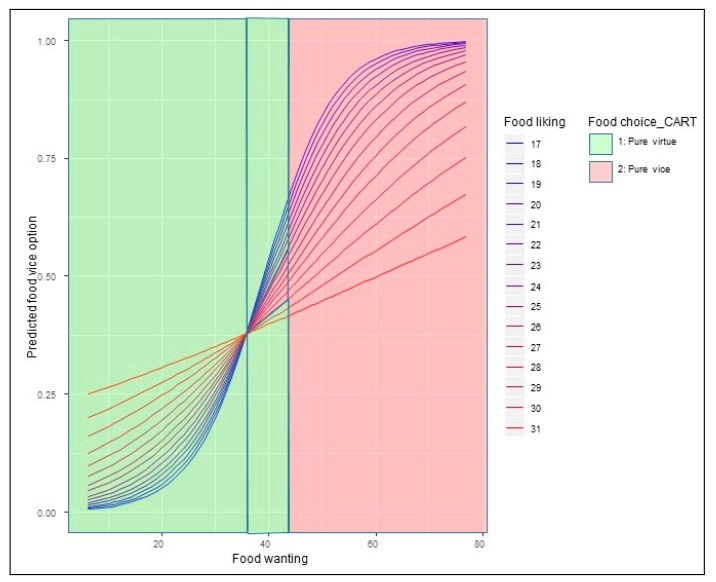
Marginal effects and Classification and Regression Tree (CART) of the interaction term (Food liking*Food wanting). Two-option set.

Hence, we also notice that the results obtained with CART match with the ones obtained with the regression analysis already presented. First, attending to the results for the two-option food choice set, we see that the classification tree shows that when the respondent reported a food wanting that scored below 36 points, the pure vice option (option 1) is the one most likely selected. These go along with the results obtained in the logistic regression: people that scored below 36 points for food wanting have a low probability of selecting the pure vice option. Above this score, it depends on how food liking and food wanting interact. At this point, we can say that CART completes the logistic regression results. The different incentive sensitivity found in logistic regression is converted into rules that allowed to predict the option selected by respondents based on their food liking and food wanting scores. Figure 10 depicts the joint results. Following the rules contained in the CART model, 63.3% of liking and food wanting interact. At this point, we can say that CART completes the logistic regression results.

**Figure 11:**
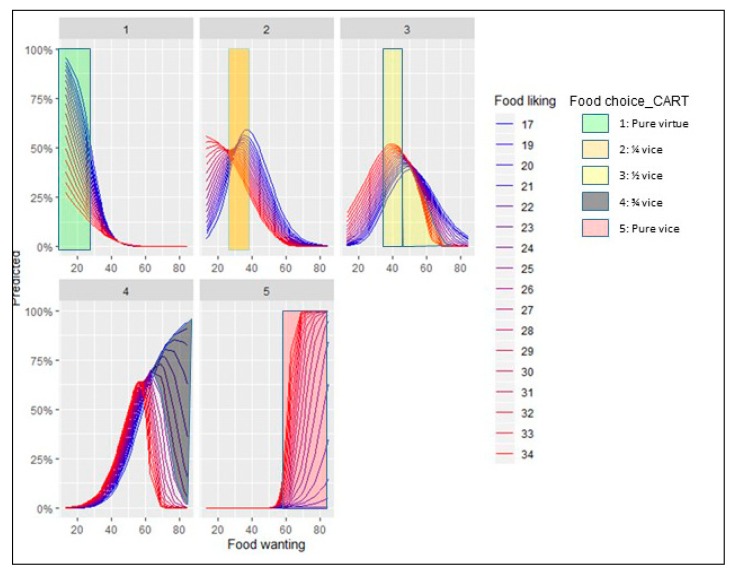
Marginal effects and CART of the interaction term (Food liking*Food wanting). Five-option set.

Second, considering the CART’s results for the five-option food choice set, we notice than they also complete the information given by the multinomial regression. Figure 11 shows the joint results obtained for multinomial regression and CART’s five-option food choice set. CART selects each option when it has the maximum probability to be chosen among the five alternatives available. When food wanting is less than 25 points, the option selected is full virtue (option 1) for any level of food liking. Option 2 (¼ food vice) is selected by people when their reported food wanting is between 25 and 36 points for any level of food liking. Option 3 (½ vice) has two different sections: for people that summed up between 36 and 47 points in food wanting this is the predicted alternative for any level of food liking; the second section of this option is predicted when interviewees scored between 47 and 59 points in food wanting and food liking was greater than 27 points. Option 4 (¾ vice) is predicted to be chosen by people that summed up between 47 and 59 points and food liking was less than 27 points (remember these were the persons who showed a higher incentive sensitivity). Finally, persons that had food wanting scores higher than 59 were predicted to elect the full vice alternative. The predictions made with the CART model for the five-option food choice set were right in 71.06 of the respondents (see plot 5 in Figure 11).

In summary, the proposed operationalization of the food liking and food wanting model trough HTAS and DEBQ selected subscales predicted accurately the food choice made by respondents with a high level of accuracy (between 63% and 71%). Food wanting is much more important than food liking for predicting food choice in almost all the cases under study. Except for the case of people that had higher incentive sensitivity, the higher the food wanting, the higher the vice content alternative selected. It caused that when more alternatives were presented to the consumer, people with higher reward profiles (‘Reward lovers’) increased the overall calories consumed. This result was caused because, even though fewer people chose the full vice option, the alternative with less vice content (pure virtue) was not selected by any of the people that belonged to this segment. It seems to be consistent with their food reward profile. In any case, the most numerous segment (‘half epicurious’) followed the expected path reducing the aggregate calories chosen when more food choice alternatives were presented. Finally, ‘Non indulgents,’ people with the lower reward profile consumed practically the same calories in both scenarios.

## 4. Discussion and Recommendations

In this study, we have seen that food pleasure (liking), food desire (wanting), and the interaction between them influence and are good predictors of food choice and food intake. Reward responses to food are closely related to food choice and may lead to caloric overconsumption. There exist different segments in the market attending to their reward-seeking attitude profile. Based on the responses given to a self-administered questionnaire measuring liking and wanting attitudes, we found three different segments named ‘Reward lovers,’ ‘Half epicurious’, and ‘Non indulgents.’ Their behavior when choosing food is quite different. ‘Vice lovers’ mainly prefer the options with more vice content, ‘Half epicurious’—placing a greater importance on addressing a health goal than a taste goal—choose options containing lower vice proportion than the before mentioned segment and, last but not least, ‘Non indulgents’ select the alternatives with more virtue. Vice-virtue bundles slightly reduce aggregate caloric consumption (−3.6%). Results show differential effects on caloric consumption depending on segments. ‘Non indulgents’ increase a little bit the calories elected (+2%), ‘Half epicurious’ reduce the caloric selection made (−9.3%), and ‘Reward lovers’ increase the calories chosen (+12.6%).

Most of the studies done using the liking-wanting paradigm belong to the neuroscience domain. Hence, the methodologies and instruments used focus on the research goals pursued, but they are difficult to apply from a managerial perspective. Our work offers an easier and more suitable instrument for practitioners to measure and manage the hedonic aspects linked to psychological factors that are part of the food choice psychobiological system. Three well-known and widely used subscales reported good reliability results for measuring the food liking (HTAS_plasure subscale) and food wanting (DEBQ_emotional and DEBQ_external subscales) consumers’ attitudes.

Our results support the initial theory that wanting and liking are associated with each other. They are significantly correlated (r = 0.56). When analyzing how they affect food choice decisions, we see that food wanting has a higher influence than food liking. Results from correlation analysis, logistic and multinomial regression confirm this postulate. Looking to the odds from the logistic and multinomial regressions, we conclude that for any unit increment in food liking and food wanting the probability of choosing an option with a higher vice component also increases. Therefore, hypotheses 1 and 2 holds.

Interaction effects between food liking and food wanting are statistically significant in both food choice sets analyzed. In the case of the two-option set, for every point on increment in the joint effect of food liking and food wanting, the probability of choosing the pure vice option decreases 1%. In the case of the five-option set, the likelihood to pick-up the pure vice alternative rises 2% for every increment point in the interaction effect. The other four options decrease 1% the probability to be chosen for every point of increment in the interaction effect. However, the interaction effect on food choice is not linear across all the levels of food liking and food wanting. To obtain a precise and more in-depth interpretation, we ran marginal effects analysis.

Results from marginal effects computed on the logit equation showed an influence of food liking-food reward interaction on the likelihood to choose the pure vice option that evolves from an S-shaped curve for the lowest levels of food liking to a rectilinear relationship for the highest levels of food liking. In practical terms, it means an increasing positive relationship for all the combinations of food liking and food wanting. In this case, we could say that hypothesis 3 holds. At first glance, the different shapes and slopes could seem counterintuitive. Why do people with less liking levels show higher sensitivity to the increments of food reward? One such circumstance can be explained through the neural sensitization theory that posits an excessive amplification of psychological wanting, mainly triggered by cues, without necessarily an amplification of ‘liking’ [17,113]. Following their proposals, for instance, a well-designed marketing campaign could trigger excessive incentive salience to food-related cues and become extra desirable. In our study, we can also see that incentive-sensitization could promote overeating episodes because the probability of selecting the pure vice option is higher when it occurs. As we said when developing hypothesis 4, unexpected encounters with cues for a reward can activate motivation to pursue that reward as a goal. A more in-depth analysis of which factors explain the found relationship between the interaction of food liking-food wanting and sensitization is needed, but is beyond the purposes of our study.

Results from marginal effects calculated on the multinomial equation showed different influence patterns of the food liking-food reward interaction on the likelihood to choose each of the five options available in the food choice set. The options with less vice content (pure virtue and ¼ vice) have higher predicted probabilities to be chosen when food liking and food wanting are lower. The three alternatives with more vice content (½ vice, ¾ vice, and full vice) have higher predicted probabilities to be elected when food liking and food wanting are higher. Consequently, we can also say that in this case, hypothesis 3 holds. Nevertheless, it has to be pointed out that for the ¾ vice option, a sensitization episode for low levels of liking and high level of wanting occurs. As in the previous case, a more in-depth analysis is necessary. Consumers with a higher incentive sensitivity have a particular interest from a marketing perspective because when the steeping point threshold of the food wanting is reached, little efforts to promote consumption trigger the probability to consume high caloric products.

When the consumer has more food-choice alternatives that combine different proportions of healthy and tasty food to decide on, the caloric content of its final selection diminishes (−3.6%). This aggregate result is in line with results obtained in previous studies [63]. Considering the odds obtained in the logistic and multinomial regression, we conclude that food liking, food wanting, and the interaction between them cause an effect that is weaker for the five-option choice set. Hence, hypotheses 5, 6, and 7 hold. Nevertheless, this assertion being true in aggregate terms, it depends on which segments we analyze.

The three segments found were formed attending to their scores on food liking and food wanting scales (‘Reward lovers’-> high liking, high wanting, ‘Half epicurious’-> medium liking, medium wanting, ‘Non indulgent’-> low liking, low wanting). Consequently, hypotheses 5, 6, and 7 must also hold for them. Calculating the calories chosen for the segments in each of the food choice sets used, we found that two segments increased the per-capita calories selected (‘Reward lovers’ +12.6% and ‘Non indulgents +2%) when the five-option food bundle was presented and only one segment reduced it (‘Half epicurious’ −9.3%). Hence, hypotheses 5, 6, and 7 only hold for the ‘Half epicurious’ segment. Analyzing the data, we observed that for ‘Reward lovers’ the most preferred options in the five-option food bundle were those with higher vice component and none of the respondents selected the pure virtue option. Therefore, although the pure vice option was less selected (2 options -> 59.3%, 5 options-> 22%), the choices with some vice summed up much more calories.

In the light of our findings, we propose that introducing vice-virtue bundles may offer an opportunity to motivate some consumers—the ones with moderate food liking and food wanting attitudes—toward a less caloric and healthier consumption. This is the majority segment found, hence in impact terms, it is worth considering. For the rest of the segments, the introduction of vice-virtue bundles did not reduce their aggregate caloric consumption. Nevertheless, consumers manifested a preference for the ¼ vice and ½ vice options over the ¾ vice options that could be explained attending to variety-seeking reasons. We must point out that our results contradict the findings of Liu et al. (2015) about the general recommendation for introducing vice-virtue bundles from a consumer welfare perspective. They suggested that vice-virtue bundles should only be introduced when the population contains a large enough percentage of initial vice choosers that there is still an aggregate saving in calories. As we have seen, the homologous segment in our study, ‘Reward lovers’, did not behave in the same way. Hence, we recommend that introducing vice-virtue bundles is a good option from the consumer welfare perspective but we need to manage special marketing efforts to persuade to ‘Rewards lovers’ (mainly) and ‘Non indulgent’ toward the less caloric choices available. In order to answer why these differences arose, we guess that different sensitivities found in the food liking-food wanting interaction term could be behind the scenes. It would be an exciting research line for the future.

For checking the robustness of our findings and introducing simplicity when managing the proposed scales, we ran a nonlinear analysis (CART). Results were robust and with a moderate to high prediction capacity (accuracy 71.06%, 10-fold cross-validation, for the five-option bundle). CART trees showed the heuristics to apply in terms of consumers’ food reward profiles using the valuation given to the food liking and food wanting scales. Thus, as it can be seen in Table 7, we found:

These results corroborate the argument that food wanting is the main determinant of food choice [17,91,92,93]. Hence, hypothesis 4 holds. Nevertheless, we would like to remember and remark the importance, and further research needed, of the liking-wanting interaction effect on food choice. As was established when formulating hypothesis 4, it would be necessary to distinguish between incentive salience wanting and ordinary wanting in order to better understand and manage the consumer’s eating behaviors. As we have seen in the results section, an incentive-sensitization or cue-triggered hyperreactivity seems to be present, causing some anomalies in the expected interaction effect among food liking and food wanting. The incentive-sensitization theory [114] could be applied to understand the internal mechanisms that connect food wanting and food choice.

Anyway, our work present several practical implications for consumers and managers. The consumers that belong to the greater segment found ‘Half epicurious’ could obtain a significant reduction in their food caloric intake when introducing food bundles with several options. The rest of the consumers must take care, mainly those that belong to the ‘Reward lovers’ segment. However, managers can help them following the recommendations derived from our findings. Companies have an opportunity to plan and implement a win-win strategy offering more variety (food choice options) and helping with a tailored marketing campaign to the consumers that are likely going to consume more calories. From a management perspective, it would be easier to manage the marketing efforts if the found segments could be described in the usual pyscho-demographic profiles. It also conforms a future line of research for practical purposes. The scales, as they have been used in our work, also seem to be suitable for all practitioners that are dealing with nutrition issues. It offers a short and quick tool (it took less than five minutes to answer to our respondents) that help to predict food choice. From a macromarketing perspective, all those institutions that deal with nutrition and health can help through social marketing campaigns to focus on reducing the sensitivity problem found mainly in consumers that belong to the ‘Reward lovers’ segment. This win-win strategy, proposed mostly in marketing terms, is an urgent necessity for our society.

In sum, food desire is more relevant than food pleasure to determine food choice, as we hypothesized. Each of the terms can be measured separately and the interaction effect between them affects differently on food choice. For the first time, our results show that this difference depends on the levels of food desire and food choice. Hence, consumer segments do not behave in the same way when more food options are available to choose from. Consumers who pursue more reward (higher food desire and food pleasure) specially need tailor-made actions, beyond increasing the number of healthier options of the food bundles, in order to balance their caloric intake.

Our study presents several limitations. First, our dataset is not rich enough in order to generalize the results obtained. Generalizing from student’s samples to the general public can be problematic when personal and attitudinal variables are used [115]. Second, it is a cross-sectional study when several investigations have demonstrated that consumers alternate between food choice options over time [116,117]. Third, our segmentation needed some pyscho-demographic variables, which were not included in the questionnaire, in order to better operationalize results in management terms. Finally, price has not been taken into account when monetary concerns are usually significant when making food choices [118].

## Figures and Tables

**Table 1:** Variables and measurements used.

*Variables*	*Measurements*
Food Pleasure	HTAS subscale of Pleasure [59]
Food Desire	The DEBQ subscales of Emotional plus External [60]
Vice- Virtue Bundles	Evenly randomized experiment of Bundle of vice-virtue options 2 (full virtue or full vice) versus 5 options concept (full virtue, ½ vice, ½ vice, ¾ vice, full vice) [63]
Food Choice	Food selection from photos of either 4 tomatoes (124 kcal) or 4 croquettes (700 kcal); or 4 tomatoes (124 kcal) 3 tomatoes and 1 croquette (268 kcal), 2 tomatoes and 2 croquettes (412 kcal), 1 tomato and 3 croquettes (556 kcal), or 4 croquettes (700 kcal)
Gender	Direct question
BMI	Direct question of weight(kg) and height(m) [101] through the formula: kg/m^2^

**Table 2:** Descriptive statistics and correlations among the variables.

	*n*	M	SD	1	2	3	4	5	6
1. Food pleasure (liking) ^a^	273	24.53	3.41	-					
2. Food desire (wanting) ^b^	273	39.85	14.89	0.56 **	-				
3. Two-options food set ^c^	138	1.42	0.49	0.22 *	0.46 **	-			
4. Five-options food set ^d^	135	2.64	1.14	0.51 **	0.77 **	^g^	-		
5. BMI ^e^	273	24.4	3.71	0.08	0.01	0.01	0.06	-	
6. Gender ^f^	273	1.62	0.48	0.12 *	0.16 **	0.04	0.03	−0.12 *	-

Notes: ** Correlation is significant at the 0.01 level (2-tailed). * Correlation is significant at the 0.05 level (2-tailed). ^a^ Coded from 0 (minimum) to 36 (maximum)-> Summing up 6 HTAS_pleasure subscale’s variables measured on a seven points Likert scale where 0 (minimum) and 6 (maximum). See Appendix B. ^b^ Coded from 0 (minimum) to 76 (maximum)-> Summing up 29 DEBQ_emotional + DEBQ_external subscales’ variables measured on a five points Likert scale where 0 (minimum) and 4 (maximum). See Appendix B. ^c^ Measured on a two options scale where 1 = Pure virtue and 2 = Pure vice. ^d^ Measured on a five options scale where 1 = Pure virtue 2 = ¼ vice 3 = ½ vice 4 = ¾ vice and 5 = Pure vice. ^e^ Calculated following the formula BMI = kg/m^2^. ^f^ Coded as 1 = Man 2 = Woman 3 = Other. ^g^ Impossible situation because it is the experiment treatment variable.

**Table 3:** Food choice option elected by respondents (percentage).

	Choice Option (% Choosing)				
Study Condition	Pure Virtue	¼ Vice	½ Vice	¾ Vice	Pure Vice
Pure virtue-pure vice (*n* = 138)	58	-	-	-	42
Vice-virtue 50/50 included (*n* = 135)	17	31	28	18	6

**Table 4:** Segment’s food choice. Pure virtue-pure vice set (calories).

		Two options			
		Pure virtue (124 Kcal.)	Pure vice (700 Kcal.)	Total (Kcal.)	Per capita (Kcal.)
Segments	Reward lovers	1116	9,800	10,916	475
	Non indulgents	6324	10,500	16,824	255
	Half epicurious	2480	20,300	27,740	566
Total		9920	40,600	50,520	366

**Table 5:** Segments food choice. Five options bundle (calories).

		Five options bundle						
		Pure virtue (124 Kcal.)	1/4 vice (268 Kcal.)	1/2 vice (412 Kcal.)	3/4 vice (556 Kcal.)	Pure vice (700 Kcal.)	Total (Kcal.)	Per capita (Kcal.)
Segments	Reward lovers	0	536	3296	5004	5600	14,436	535
	Non indulgents	2108	6164	4944	556	0	13,772	260
	Half epicurious	744	4556	7416	7784	0	28,208	513
Total		2852	11,256	15,656	13,344	5600	48,708	361

**Table 6:** Logistic and multinomial regression results. (food choices vs. food liking + food wanting).

	Dependent Variable									
	Two-Choices -Logit- (Ref.Category = Pure Virtue)		Five-Choices -Multinomial- (Ref.Category = Pure Virtue)							
	Pure Vice		1/4 Vice		1/2 Vice		3/4 Vice		Pure Vice	
	Log-odds	Odds	Log-odds	Odds	Log-odds	Odds	Log-odds	Odds	Log-odds	Odds
Food liking	0.336 (0.212)	1.4	0.346 *** (0.057)	1.41	0.438 *** (0.068)	1.55	0.430 *** (0.088)	1.53	−1.235 *** (0.391)	0.29
Food wanting	0.312 ** (0.128)	1.37	0.351 *** (0.086)	1.42	0.430 *** (0.091)	1.54	0.511 *** (0.098)	1.67	−0.031 (0.175)	0.97
Food liking * Food wanting	−0.009 * (0.005)	0.99	−0.009 *** (0.003)	0.99	−0.009 *** (0.004)	0.99	−0.08 ** (0.004)	0.99	0.025 ** (0.011)	1.02
Intercept	−11.648 ** (5.228)	0.00	−11.537 ** (0.004)	0.00	−16.811 *** (0.004)	0.00	−21.988 *** (0.004)	0.00	−0.598 *** (0.016)	0.55
Akaike Information Criteria	160.1		305.2		305.2		305.2		305.2	

Note: * *p* < 0.05, ** *p* <0.01, *** *p* <0.001

**Table 7:** Heuristic rules for predicting food choice considering food reward attitudes.

Option	Heuristic Rule
Pure virtue	Food wanting < 25
¼ vice	Food wanting 26–36
½ vice	Food wanting 36–47 Food wanting 47–59 and food liking > 27
¾ vice	Food wanting 47–59 and food liking < 27
Full vice	Food wanting > 59

## Appendix B

### Reliability Analysis

**Table A1:** Means, standard deviations (SD), and reliabilities for used sub-scales.

	Mean	SD	Factor Loading	Communality	Item-Total Correlation
*HTAS_pleasure (α = 0.74, Variance% = 44.7)*					
1. I do not believe that food should always be source of pleasure.	3.9	1.4	0.79	0.63	0.7
2. The appearance of food makes no difference to me.	5.0	1.2	0.69	0.48	0.6
3. When I eat, I concentrate on enjoying the taste of food.	4.6	1.5	0.59	0.35	0.6
4. It is important for me to eat delicious food on weekdays as well as weekends.	4.9	1.1	0.56	0.31	0.5
5. An essential part of my weekend is eating delicious food.	3.6	1.7	0.51	0.26	0.6
6. I finish my meal even when I do not like the taste of a food.	4.6	2.0	0.81	0.66	0.9
*DEBQ_emotional (α = 0.93,Variance% = 55.4 *)*					
7. Do you have the desire to eat when you are irritated?	1.4	1.1	0.67	0.48	0.6
8. Do you have a desire to eat when you have nothing to do?	2.2	1.1	0.55	0.84	0.6
9. Do you have a desire to eat when you are depressed or discouraged?	1.8	1.2	0.75	0.61	0.7
10. Do you have a desire to eat when you are feeling lonely?	1.5	1.1	0.67	0.62	0.7
11. Do you have a desire to eat when somebody lets you down?	1.0	1.1	0.82	0.68	0.8
12. Do you have a desire to eat when you are cross?	1.0	1.0	0.82	0.69	0.7
13. Do you have a desire to eat when you are approaching something unpleasant to happen?	0.9	1.0	0.77	0.62	0.7
14. Do you get the desire to eat when you are anxious, worried or tense?	1.6	1.3	0.74	0.58	0.7
15. Do you have a desire to eat when things are going against you or when things have gone wrong?	1.3	1.2	0.86	0.74	0.8
16. Do you have a desire to eat when you are frightened?	0.6	0.8	0.70	0.55	0.6
17. Do you have a desire to eat when you are disappointed?	1.0	1.0	0.84	0.72	0.8
18. Do you have a desire to eat when you are emotionally upset?	1.1	1.0	0.83	0.69	0.8
19. Do you have a desire to eat when you are bored or restless?	1.9	1.2	0.57	0.76	0.6
*DEBQ_external (α = 0.79, Variance% = 43 **)*					
20. If food tastes good to you, do you eat more than usual?	2.6	1.0	0.80	0.62	0.6
21. If food smells and looks good, do you eat more than usual?	2.6	1.0	0.84	0.64	0.7
22. If you see or smell something delicious, do you have a desire to eat it?	3.0	0.9	0.80	0.63	0.6
23. If you have something delicious to eat, do you eat it straight away?	2.4	1.0	0.71	0.54	0.5
24. If you walk past the baker do you have the desire to buy something delicious?	2.2	1.1	0.49	0.61	0.6
25. If you walk past a snackbar or a cafe, do you have the desire to buy something delicious?	1.7	1.1	0.32	0.53	0.7
26. If you see others eating, do you also have the desire to eat?	1.8	1.1	0.54	0.61	0.6
27. Can you resist eating delicious foods?	2.1	1.1	-0.36	-0.29	0.1
28. Do you eat more than usual, when you see others eating?	1.4	1.1	0.37	0.50	0.5
29. When preparing a meal are you inclined to eat something?	2.9	1.1	0.56	0.34	0.3

Notes: * *QUARTIMAX rotation ran in IBM-SPSS version 25*. ** *OBLIMIN rotation (delta = 0) ran in IBM-SPSS version 25*.

## Appendix D

### Segments’ Food Choice

**Table A2:** Segment’s food choice. Pure virtue-pure vice set (number of respondents).

		Two options		Total
		Pure virtue	Pure vice	
Segments	Reward lovers	11	16	27
	Half epicurious	34	36	70
	Non indulgents	35	6	41
Total		80	58	138

**Table A3:** Segments food choice. Five options bundle (number of respondents).

		Five options bundle					
		Pure virtue	¼ vice	½ vice	¾ vice	Pure vice	Total	
Segments	Reward lovers	0	4	12	12	8	36	
	Half epicurious	9	27	24	12	0	72	
	Non indulgents	14	11	2	0	0	27	
Total		23	42	38	24	8	135	

## Appendix E

### ANOVA Analysis

**Table A4:** ANOVA tests results on Food choice as a function of Bundles and Segments.

Dependent Variable: Food Choice								
Source	Type III Sum of Squares	df	Mean Square	F	Sig.	Partial Eta Squared	Noncent. Parameter	Observed Power ^b^
Corrected Model	142.436 ^a^	5	28.487	13.378	0.000	0.200	66.889	1.000
Intercept	1611.914	1	1611.914	756.966	0.000	0.739	756.966	1.000
Bundles	0.401	1	0.401	0.189	0.664	0.001	0.189	0.072
Segments	125.534	2	62.767	29.476	0.000	0.181	58.952	1.000
Bundles * Segments	7.795	2	3.898	1.830	0.162	0.014	3.661	0.380
Error	568.561	267	2.129					
Total	2647.000	273						
Corrected Total	710,996	272						

^a^ R Squared = 0.200 (Adjusted R Squared = 0.185). ^b^ Computed using alpha = 0.05.
